# Modeling of Sensor Placement Strategy for Shape Sensing and Structural Health Monitoring of a Wing-Shaped Sandwich Panel Using Inverse Finite Element Method

**DOI:** 10.3390/s17122775

**Published:** 2017-11-30

**Authors:** Adnan Kefal, Mehmet Yildiz

**Affiliations:** 1 Composite Technologies Center of Excellence, Istanbul Technology Development Zone, Sabanci University-Kordsa Global, Pendik, Istanbul 34906, Turkey; meyildiz@sabanciuniv.edu; 2 Integrated Manufacturing Technologies Research and Application Center, Sabanci University, Tuzla, Istanbul 34956, Turkey; 3 Faculty of Engineering and Natural Sciences, Sabanci University, Tuzla, Istanbul 34956, Turkey

**Keywords:** inverse finite element method (iFEM), refined zigzag theory (RZT), shape-sensing, sandwich plate, structural health monitoring, aerospace structures

## Abstract

This paper investigated the effect of sensor density and alignment for three-dimensional shape sensing of an airplane-wing-shaped thick panel subjected to three different loading conditions, i.e., bending, torsion, and membrane loads. For shape sensing analysis of the panel, the Inverse Finite Element Method (iFEM) was used together with the Refined Zigzag Theory (RZT), in order to enable accurate predictions for transverse deflection and through-the-thickness variation of interfacial displacements. In this study, the iFEM-RZT algorithm is implemented by utilizing a novel three-node C°-continuous inverse-shell element, known as i3-RZT. The discrete strain data is generated numerically through performing a high-fidelity finite element analysis on the wing-shaped panel. This numerical strain data represents experimental strain readings obtained from surface patched strain gauges or embedded fiber Bragg grating (FBG) sensors. Three different sensor placement configurations with varying density and alignment of strain data were examined and their corresponding displacement contours were compared with those of reference solutions. The results indicate that a sparse distribution of FBG sensors (uniaxial strain measurements), aligned in only the longitudinal direction, is sufficient for predicting accurate full-field membrane and bending responses (deformed shapes) of the panel, including a true zigzag representation of interfacial displacements. On the other hand, a sparse deployment of strain rosettes (triaxial strain measurements) is essentially enough to produce torsion shapes that are as accurate as those of predicted by a dense sensor placement configuration. Hence, the potential applicability and practical aspects of i3-RZT/iFEM methodology is proven for three-dimensional shape-sensing of future aerospace structures.

## 1. Introduction

Structural health monitoring (SHM) has become a critical process for composite structures, particularly those used in aeronautical, naval and civil applications [1,2,3]. The implementation of SHM to composite structures can enable the acquisition of real-time deformation and stress information by utilizing in situ sensors. This is generally referred to as “shape and stress sensing”, and can be potentially useful for predicting real-time damage (i.e., unhealthy structural conditions) and even to evaluate fatigue life. Therefore, the safety and structural integrity of a structure can be maintained at reduced repair cost. 

Various researchers have utilized an SHM system to investigate different types of failure phenomena of composites, including delamination [4], face/core debonding [5], and fatigue responses [6], and failure modes of foam core sandwich composites [7]. Moreover, shape sensing of a morphing (smart) wing [8] and damage detection of wind turbine blades [9] was demonstrated by taking advantage of embedded optical-fiber networks, such as fiber Bragg grating (FBG) sensors. In addition, the feasibility of manufacturing composite laminates with built-in FBG sensors have been elaborated on [10] for collecting a large amount of strain data. Furthermore, various real-time monitoring algorithms have been proposed for beam and plate bending. These methods include modal-based approaches [11,12,13], curve-fitting and regression algorithms [14,15,16,17], Tikhonov’s [18] regularization techniques [19,20], and least-squares algorithms [21,22,23]. Nevertheless, the complexity of boundary conditions and structural topology were not considered by the most of these shape sensing methods. Therefore, they may not be used as a general-purpose on-site SHM algorithm. We elaborate on this issue in the following four paragraphs by providing a brief background on these methods.

The modal-based inverse algorithms use vibration mode shapes of the body and experimental strain measurements (e.g., strain gauge readings) to construct the displacement-strain relationship. Although the effectiveness of these algorithms was demonstrated for a clamped end beam [11] and cantilever plates [12,13], the number of estimated mode shapes was only restricted to the number of strain sensors placed on the structures, thus requiring more sensors to estimate the vibration or deformed shapes at higher frequency excitations.

The curve-fitting and regression algorithms mainly aim to fit experimentally measured strains into an a priori set of global and piece-wise continuous basis functions and proper weights. Then, the displacement field of the beam/plate structure can be evaluated, utilizing strain-displacement relationships. The example applications of these methods to simple beam structures can be found in [14,15,16]. In particular, Ko et al. [17] computed the deflection and cross-section twist of an aircraft wing, using a load-independent method that approximates the beam curvature with piece-wise polynomials. However, curve-fitting algorithms may require many trial functions and strain sensors for the reconstruction of more complicated deformations.

Tikhonov’s [18] regularization can guarantee a confident smoothness degree, to solve the inverse problem of shape sensing. This type of regularization technique was utilized to solve inverse elastic problems [19] and to calculate surface tractions on a body, from internal displacements measured at discrete sensor locations [20]. The spatial regularization technique was also employed concurrently with a statistical approach, to estimate the errors in the solution of an inverse problem [21]. However, this methodology requires iterations and may therefore lead to convergence difficulties and high computational costs, especially for complex three-dimensional structures.

A least-squares formulation [22] was utilized to solve the shape-sensing problem of a cantilever plate, based on the assumptions of Kirchhoff plate hypotheses. However, this formulation is not general enough for complex geometries, due to the inherent assumptions made for a simple cantilever plate. Another shape-reconstruction algorithm [23] proposed, based on a weighted-least-squares functional, aimed to enforce the compatibility between the analytical and measured bending curvatures of the Kirchhoff plate theory. Nevertheless, it is difficult to generalize this approach, since the weighting coefficients in the least-square terms are computed to resolve inherent errors in the strain-sensor data, by considering the given data-acquisition tool, the load condition, and the test specimen. Furthermore, an inverse interpolation algorithm, involving least-squares minimization of calculated and measured strains, was proposed for shape sensing of aerospace structures [24]. However, this methodology requires the recovery of the applied loading before the solution of displacements, but the loading conditions may have a non-trivial physical topology in the real environment, thus causing the main drawback of the proposed formulation.

An original mechanics-based algorithm called the “Inverse Finite Element Method (iFEM)” was developed by Tessler and Spangler [25,26], for shape sensing of plate and shell structures. The mathematical foundation is based on minimization of a least-squares functional, defined by the summation of squared errors between analytical and experimental strain measures. In comparison to other shape-sensing methods, iFEM serves an advantage for estimating static and dynamic behavior of any structural topology with any constraint boundary condition. The second advantage of iFEM is that no prior knowledge of loading and material is required for shape sensing analysis. Finally, iFEM has a robust formulation, which makes it suitable for real-time monitoring applications.

The iFEM approach has been studied extensively over the past fifteen years. To give some pioneering examples, various types of inverse elements have been developed, including a three-node triangle [27] and a four-node quadrilateral [28] inverse-shell element, and a viable inverse beam/frame element [29,30]. Other examples include the practical applications of iFEM framework to SHM of aerospace vehicles [31,32,33], marine structures [34,35] including chemical tankers [36], containerships [37], bulk carriers [38], and offshore structures [39]. Also, a US patent was obtained to perform shape sensing of downhole structures [40]. Recently, Cerracchio et al. [41] developed an iFEM framework, based on the kinematic assumptions of the Refined Zigzag Theory (RZT) [42], in order to account for relatively thick sandwich plates. Then, Kefal and co-workers [43] enhanced the iFEM-RZT formulation and developed a novel three-node C^0^-continuous inverse-shell element (i3-RZT). This improved iFEM-RZT formulation with the i3-RZT element [43] is well suited to the real-time displacement and stress monitoring of thin, moderately thick, and thick composite shell structures that are instrumented with few strain sensors.

Based on the enhanced iFEM-RZT [43] methodology, this study investigates the effect of sensor density and alignment on the shape sensing of a tapered wing-shaped sandwich panel, subjected to membrane, bending, and torsion loads, for the first time in the literature. This paper is structured as follows: First of all, the theoretical basis of the iFEM-RZT and implementation of the i3-RZT element is briefly reviewed in the remainder of the paper. Then, a high-fidelity FEM analysis of the sandwich plate is described, which generated the discrete strain data to represent experimental strain measurements collected from in situ strain gauges/rosettes and FBG sensors. After that, the performance of three different sensor placement configurations, including dense, sparse, and very sparse sensor distributions, which formed on the sandwich panel, and the shape sensing analysis, using i3-RZT elements, is described. Finally, the most efficient and practical sensor network is determined for each loading scenario, by comparing iFEM-RZT displacement results with those of FEM analyses. 

## 2. The Enhanced iFEM-RZT Formulation

### 2.1. The i3-RZT Inverse-Shell Element

**Figure 1:**
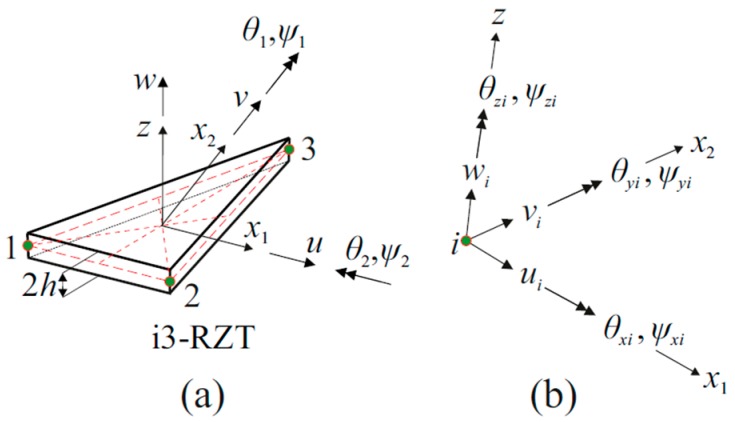
(**a**) The three-node triangular inverse-shell element (i3-RZT) element, depicted with its local coordinate system; (**b**) nodal degrees-of-freedom in the local coordinate system.

The three-node triangular inverse-shell element, i3-RZT, developed by Kefal et al. [43] will be briefly reviewed, to demonstrate iFEM-RZT formulation. To define the displacement and strain fields of the element, an orthogonal local coordinate system $\left(x_{1},x_{2},z\right)$ is chosen and its origin (0,0,0) is positioned at the centroid of the mid-plane triangle, as shown in Figure 1a. Note that, the coordinates $x\equiv (x_{1},x_{2})$ are in-plane coordinates and z∈[−h,+h] defines the thickness coordinate. The element has nine displacement degrees-of-freedom (DOF) at each corner node, as depicted in Figure 1b. These nodal DOF consist of positive $x_{1}$, $\;x_{2}$, z translations $u_{i}$, $v_{i}$, $w_{i}$, positive counter clockwise classical rotations around the $x_{1}$- and $x_{2}$-axes, $\theta_{xi}$ and $\theta_{yi}$, positive counter clockwise zigzag rotations around the $x_{1}$- and $x_{2}$-axes, $\psi_{xi}$ and $\psi_{yi}$, drilling rotations $\theta_{zi}$, and artificial zigzag rotations $\psi_{zi}$. When modelling built-up or curved shell structures use the i3-RZT element, the element can simply avoid singular solutions as a result of the presence of $\theta_{zi}$ and $\psi_{zi}$.

The membrane displacements along the $x_{1}$ and $x_{2}$ directions, u(x) and v(x), can be defined in terms of the nodal DOF of $u_{i}$, $v_{i}$ and $\theta_{zi}$ as

(1a) $$u(x)\equiv u=\sum_{i=1}^{3}\left(N_{i}\;u_{i}+L_{i}\;\theta_{zi}\right)$$

(1b) $$v(x)\equiv v=\sum_{i=1}^{3}\left(N_{i}\;v_{i}+M_{i}\;\theta_{zi}\right)$$

In addition, the transverse deflection w(x), bending and zigzag rotations around the positive $x_{2}$ and negative $x_{1}$ directions, $\chi_{1}(x)$ and $\chi_{2}(x)$ (χ=θ,ψ), can be expressed by the nodal DOF of $w_{i}$, $\theta_{\alpha i}$ and $\psi_{\alpha i}$ (α=x,y) as

(2a) $$w(x)\equiv w=\sum_{i=1}^{3}\left[N_{i}\;w_{i}-L_{i}(\theta_{xi}-\psi_{xi})-M_{i}(\theta_{yi}-\psi_{yi})\right]$$

(2b) $$\chi_{1}(x)\equiv \chi_{1}=\sum_{i=1}^{3}N_{i}\;\chi_{y}{}_{i}\;\;\;\;\;(\chi =\theta ,\psi )$$

(2c) $$\chi_{2}(x)\equiv \chi_{2}=-\sum_{i=1}^{3}N_{i}\;\chi_{x}{}_{i}\;\;\;\;\;(\chi =\theta ,\psi )$$

where $N_{i}$ is the linear area-parametric coordinates of the triangle and $L_{i}$ and $M_{i}$ are the anisoparametric interpolation functions that are explicitly given in Appendix A. Note that these shape functions are obtained by applying Tessler–Dong’s constant shear edge constraint conditions [44,45] and they were originally used in [46].

For a laminate with N perfectly bonded laminae, RZT establishes in-plane displacement components $u_{\alpha }^{\left(k\right)}(x,z)\;\;(\alpha =1,2)$ of any material point as functions of constant, linear, and zigzag variations of the thickness coordinate as [42]:

(3a) $$u_{1}^{\left(k\right)}(x,z)\equiv u_{1}^{\left(k\right)}=u+z\theta_{1}+\phi_{1}^{\left(k\right)}\;\psi_{1}$$

(3b) $$u_{2}^{\left(k\right)}(x,z)\equiv u_{2}^{\left(k\right)}=v+z\theta_{2}+\phi_{2}^{\left(k\right)}\;\psi_{2}$$

and constant transverse displacement through the thickness of the laminate as:

(3c) $$u_{z}(x,z)\equiv u_{z}=w$$

**Figure 2:**
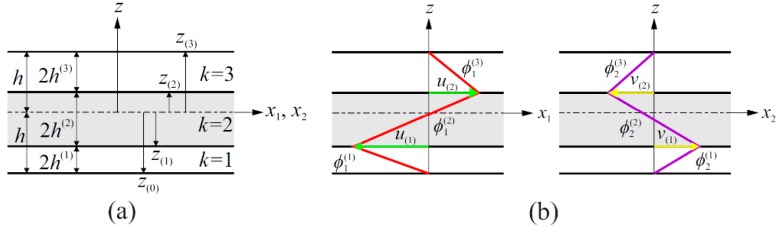
(**a**) Layer notation for a three-layer laminate; (**b**) through-the-thickness piecewise-linear zigzag functions.

As described in Figure 2a, the superscript (*k*) used in Equation (3a,b) indicates the *k*-th lamina, whereas the subscript (*k*) defines the interface between the *k*-th and (*k* + 1)-th laminae. Moreover, as depicted in Figure 2b, the functions $\phi_{1}^{\left(k\right)}\equiv \phi_{1}^{\left(k\right)}(z)$ and $\phi_{2}^{\left(k\right)}\equiv \phi_{2}^{\left(k\right)}(z)$ denote through-the-thickness piecewise-linear zigzag functions that can be expressed as [42]:

(4a) $$\phi_{1}^{\left(k\right)}\equiv \frac{1}{2}(1-\xi^{\left(k\right)})\;u_{\left(k-1\right)}+\frac{1}{2}(1+\xi^{\left(k\right)})\;u_{\left(k\right)}$$

(4b) $$\phi_{2}^{\left(k\right)}\equiv \frac{1}{2}(1-\xi^{\left(k\right)})\;v_{\left(k-1\right)}+\frac{1}{2}(1+\xi^{\left(k\right)})\;v_{\left(k\right)}$$

with

(4c) $$\xi^{\left(k\right)}=\left[\frac{z-z_{\left(k-1\right)}}{h^{\left(k\right)}}-1\right]\in [-1,+1]\;\;\;\;(k=1,2,\ldots ,N)$$

where the first lamina, beginning at $z_{\left(0\right)}=-h$, the last (*N*-th) lamina ending at $z_{\left(N\right)}=+h$, and the *k*-th lamina, ending at $z_{\left(k\right)}=z_{\left(k-1\right)}+2h^{\left(k\right)}$, where $2h^{\left(k\right)}$ denotes the thickness of the *k*-th lamina. Calculating the zigzag functions at the lamina interfaces, $z=z_{\left(k\right)}$ and $z=z_{\left(k-1\right)}$, gives rise to the definition of interfacial displacements as (see Figure 2b):

(5a) $$\begin{array}{l} u_{\left(k\right)}=\phi_{1}^{\left(k\right)}(\xi^{\left(k\right)}=+1),\;v_{\left(k\right)}=\phi_{2}^{\left(k\right)}(\xi^{\left(k\right)}=+1)\;\;\;\;\;\;\;\;(z=z_{\left(k\right)})\\ u_{\left(k-1\right)}=\phi_{1}^{\left(k\right)}(\xi^{\left(k\right)}=-1),\;v_{\left(k-1\right)}=\phi_{2}^{\left(k\right)}(\xi^{\left(k\right)}=-1)\;\;\;\;\;(z=z_{\left(k-1\right)}) \end{array}$$

According to RZT [42], the zigzag functions at the bottom $\left(z=z_{\left(0\right)}=-h\right)$ and top $\left(z=z_{\left(N\right)}=+h\right)$ plate surfaces vanish identically; thus, interfacial displacements at these bounding surfaces can be calculated, in accordance with the Equation (5a) as:

(5b) $$u_{\left(0\right)}=u_{\left(N\right)}=v_{\left(0\right)}=v_{\left(N\right)}=0$$

Besides, the $u_{\left(k\right)}$ and $v_{\left(k\right)}$, the interfacial values of the zigzag functions can be expressed in terms of piecewise constant slope functions $\beta_{\alpha }^{\left(k\right)}\;\;(\alpha =1,2\;;\;\;k=1,2,\ldots ,N)$ as [42]:

(6a) $$\left\{\begin{array}{c} u_{\left(k\right)}\\ v_{\left(k\right)} \end{array}\right\}=2h^{\left(k\right)}\left\{\begin{array}{c} \beta_{1}^{\left(k\right)}\\ \beta_{2}^{\left(k\right)} \end{array}\right\}+\left\{\begin{array}{c} u_{\left(k-1\right)}\\ v_{\left(k-1\right)} \end{array}\right\}\;\;\;\;(k=1,2,\ldots ,N)$$

where the $\beta_{\alpha }^{\left(k\right)}\;\;(\alpha =1,2)$ slope of the zigzag functions, i.e., $\partial \phi_{\alpha }^{\left(k\right)}/\partial z\;\;\;(\alpha =1,2)$, can be explicitly defined for the *k*-th layer as:

(6b) $$\left\{\begin{array}{c} \beta_{1}^{\left(k\right)}\\ \beta_{2}^{\left(k\right)} \end{array}\right\}=\left\{\begin{array}{c} G_{1}/Q_{11}^{\left(k\right)}-1\\ G_{2}/Q_{22}^{\left(k\right)}-1 \end{array}\right\}\;\;\;\;(k=1,2,\ldots ,N)$$

with the weighted-average transverse-shear stiffness coefficients, $G_{1}$ and $G_{2}$, that can be defined in terms of their respective lamina-level coefficients, $Q_{11}^{\left(k\right)}$ and $Q_{22}^{\left(k\right)}$ as:

(6c) $$\left\{\begin{array}{c} G_{1}\\ G_{2} \end{array}\right\}=\left\{\begin{array}{c} {\left(\frac{1}{h}\sum_{i=1}^{N}\frac{h^{\left(i\right)}}{Q_{11}^{\left(i\right)}}\right)}^{-1}\\ {\left(\frac{1}{h}\sum_{i=1}^{N}\frac{h^{\left(i\right)}}{Q_{22}^{\left(i\right)}}\right)}^{-1} \end{array}\right\}$$

From calculating relevant derivatives of Equation (3), the linear strain-displacement relations give rise to the in-plane and transverse-shear strain components, defined as:

(7a) $$\left\{\begin{array}{c} \varepsilon_{11}^{\left(k\right)}\\ \varepsilon_{22}^{\left(k\right)}\\ \gamma_{12}^{\left(k\right)} \end{array}\right\}=\left\{\begin{array}{c} u_{1,1}^{\left(k\right)}\\ u_{2,2}^{\left(k\right)}\\ u_{1,2}^{\left(k\right)}+u_{2,1}^{\left(k\right)} \end{array}\right\}\equiv e(u^{e})+z\kappa (u^{e})+\mu^{\left(k\right)}(u^{e})$$

and

(7b) $$\left\{\begin{array}{c} \gamma_{1z}^{\left(k\right)}\\ \gamma_{2z}^{\left(k\right)} \end{array}\right\}\equiv \left\{\begin{array}{c} u_{1,z}^{\left(k\right)}+u_{z,1}\\ u_{2,z}^{\left(k\right)}+u_{z,2} \end{array}\right\}=H_{\beta }^{\left(k\right)}\left\{\begin{array}{c} \gamma (u^{e})\\ \eta (u^{e}) \end{array}\right\}$$

with

(7c) $$H_{\beta }^{\left(k\right)}=\left[\begin{array}{cccc} 1+\beta_{1}^{\left(k\right)} & 0 & -\beta_{1}^{\left(k\right)} & 0\\ 0 & 1+\beta_{2}^{\left(k\right)} & 0 & -\beta_{2}^{\left(k\right)} \end{array}\right]$$

where the symbols, ${\left(\cdot \right)}_{,\alpha }\equiv \frac{\partial (\cdot )}{\partial x_{\alpha }}$ and ${\left(\cdot \right)}_{,z}\equiv \frac{\partial (\cdot )}{\partial z}$, signify a partial derivative, with respect to the in-plane coordinates, $x_{\alpha }\;\;(\alpha =1,2)$, and thickness coordinate, z, respectively. In Equation (7a), the vectors $e(u^{e})$, $\kappa (u^{e})$ and $\mu^{\left(k\right)}(u^{e})$ indicate the membrane strain measures, bending curvatures, and zigzag strain measures, respectively. On the other hand, the vectors, $\gamma (u^{e})$ and $\eta (u^{e})$, defined in Equation (7b), represent the first (average) and second transverse-shear strain measures, respectively. After introducing Equations (1) and (2) into Equation (7a,b), these strain measures can be explicitly defined in terms of nodal displacement vector of i3-RZT, $u^{e}$, as

(8a) $$e(u^{e})={\left[\begin{array}{ccc} u_{,1} & v_{,2} & u_{,2}+v_{,1} \end{array}\right]}^{T}=B^{e}u^{e}$$

(8b) $$\kappa (u^{e})={\left[\begin{array}{ccc} \theta_{1,1} & \theta_{2,2} & \theta_{1,2}+\theta_{2,1} \end{array}\right]}^{T}=B^{\kappa }u^{e}$$

(8c) $$\mu^{\left(k\right)}(u^{e})={\left[\begin{array}{ccc} \phi_{1}^{\left(k\right)}\psi_{1,1} & \phi_{2}^{\left(k\right)}\psi_{2,2} & \phi_{1}^{\left(k\right)}\psi_{1,2}+\phi_{2}^{\left(k\right)}\psi_{2,1} \end{array}\right]}^{T}=H_{\phi }^{\left(k\right)}B^{\mu }u^{e}$$

(8d) $$\gamma (u^{e})\equiv {\left[\begin{array}{cc} \gamma_{1} & \gamma_{2} \end{array}\right]}^{T}\equiv {\left[\begin{array}{cc} w_{,1}+\theta_{1} & w_{,2}+\theta_{2} \end{array}\right]}^{T}=B^{\gamma }u^{e}$$

(8e) $$\eta (u^{e})\equiv {\left[\begin{array}{cc} \gamma_{1}-\psi_{1} & \gamma_{2}-\psi_{2} \end{array}\right]}^{T}=B^{\eta }u^{e}$$

with

(8f) $$H_{\phi }^{\left(k\right)}=\left[\begin{array}{cccc} \phi_{1}^{\left(k\right)} & 0 & 0 & 0\\ 0 & \phi_{2}^{\left(k\right)} & 0 & 0\\ 0 & 0 & \phi_{1}^{\left(k\right)} & \phi_{2}^{\left(k\right)} \end{array}\right]$$

and

(8g) $$u^{e}={\left[\begin{array}{ccc} u_{1}^{e} & u_{2}^{e} & u_{3}^{e} \end{array}\right]}^{T}$$

(8h) $$u_{i}^{e}={\left[\begin{array}{ccccccccc} u_{i} & v_{i} & w_{i} & \theta_{xi} & \theta_{yi} & \theta_{zi} & \psi_{xi} & \psi_{yi} & \psi_{zi} \end{array}\right]}^{T}(i=1,2,3)$$

where the matrices $B^{\alpha }\;\;(\alpha =e,\kappa ,\mu ,\gamma ,\eta )$ contain derivatives of the shape functions (refer to Appendix A).

### 2.2. In Situ Section Strains

**Figure 3:**
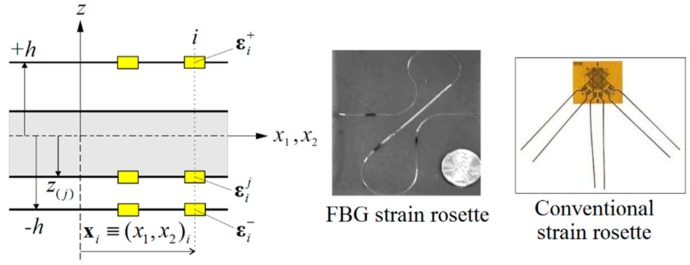
Strain rosettes and experimental surface strain measurements.

As shown in Figure 3, three different conventional strain gauges and/or embedded FBG sensors can be stacked together to form a strain rosette for gathering onboard triaxial strain measurements. Based on the iFEM-RZT methodology, three different strain rosettes $\left(\varepsilon_{i}^{+},\varepsilon_{i}^{-},\varepsilon_{i}^{j}\right)$ should be located along the thickness direction of each particular location $\left(x_{i},z)\;(i=1,2,\ldots ,n\right)$, where ‘n’ indicates the total number of discrete sensor locations. For example, conventional strain rosettes can be pathed on the bounding surface of the laminate, while FBG strain rosettes can be embedded inside the laminate, as depicted in Figure 3. 

The strain readings of these rosettes can be expressed as:

(9a) $$\varepsilon_{i}^{+}={\left[\begin{array}{ccc} \varepsilon_{11}^{+} & \varepsilon_{22}^{+} & \gamma_{12}^{+} \end{array}\right]}_{i}^{T}$$

(9b) $$\varepsilon_{i}^{-}={\left[\begin{array}{ccc} \varepsilon_{11}^{-} & \varepsilon_{22}^{-} & \gamma_{12}^{-} \end{array}\right]}_{i}^{T}$$

(9c) $$\varepsilon_{i}^{j}={\left[\begin{array}{ccc} \varepsilon_{11}^{j} & \varepsilon_{22}^{j} & \gamma_{12}^{j} \end{array}\right]}_{i}^{T}$$

where the strain measurements with subscripts (11), (22) and (12) refer to as normal strains along $x_{1}$ and $x_{2}$ directions and shear strain in the $x_{1}x_{2}$ plane, respectively. Note that, also, the superscripts ‘+’, ‘−’ and ‘*j*’ denote the strain rosettes located on the top, bottom, and *j*-th interface of the laminate, respectively.

Since the zigzag functions on the top and bottom surfaces disappear, in situ membrane strains and bending curvatures can be computed utilizing the original iFEM plate formulation [26] as:

(10a) $${Ε}_{i}=\frac{1}{2}\left(\varepsilon_{i}^{+}+\varepsilon_{i}^{-}\;\right)\;\;\;\;\;\;(i=1,2,\ldots ,n)$$

(10b) $${Κ}_{i}=\frac{1}{2h}\left(\varepsilon_{i}^{+}-\varepsilon_{i}^{-}\;\right)\;\;\;\;\;\;(i=1,2,\ldots ,n)$$

where in situ section strains, $E_{i}$ and $K_{i}$, correspond to their analytic counterparts, $e(u^{e})$ and $\kappa (u^{e})$, given by Equation (7a), respectively. Also, at a particular discrete location $\left(x_{i},z_{i}=z_{\left(j\right)}\right)$, the experimental zigzag strains can be calculated in accordance with Cerracchio et al. [41] as:

(11) $${Μ}_{i}^{j}=\varepsilon_{i}^{j}-E_{i}\;-z_{\left(j\right)}{Κ}_{i}\;\;\;\;\;\;\;\;(i=1,2,\ldots ,n)$$

Note that it is necessary to compute the analytic counterpart $\mu^{\left(k\right)}(u^{e})$ of in situ section strains $M_{i}^{j}$ at exactly the same *j*-th interface. In contrast to experimental in-plane section strains, experimental counterparts of $\gamma (u^{e})$ and $\eta (u^{e})$ (i.e., the in situ first and second transverse-shear strains $\Gamma_{i}$ and ${Η}_{i}$) cannot be directly calculated using surface strain readings $\left(\varepsilon_{i}^{+},\varepsilon_{i}^{-},\varepsilon_{i}^{j}\right)$. Kefal et al. [43] established a computational procedure to predict these in situ strain measures, by using smoothing element analysis (SEA) [47,48] and governing (equilibrium) equations of RZT. This computational tool can be useful for treating problems exhibiting larger transverse-shear strains, such as thick composite plates/shells. For thin composite plates/shells, nevertheless, the in-plane strain components contribute to the deformation much more than transverse-shear strain components. Thus, the $\Gamma_{i}$ and ${Η}_{i}$ contributions can be safely neglected during an iFEM analysis of thin shells. 

### 2.3. The Weighted-Least-Squares Functional

For an individual i3-RZT element, the analytical and experimental strains associated with the membrane, bending, zigzag, and transverse-shear deformations can be interrelated based on a weighted-least-squares functional $\Phi_{e}(u^{e})$ as [43]

(12) $$\begin{array}{ll} \Phi_{e}(u^{e})= & w_{e}{\left\|e(u^{e})-Ε\right\|}^{2}+w_{\kappa }{\left\|\kappa (u^{e})-Κ\right\|}^{2}+w_{\mu }{\left\|\mu^{\left(k\right)}(u^{e})-{Μ}^{j}\right\|}^{2}\\ & +w_{\gamma }{\left\|\gamma (u^{e})-\Gamma \right\|}^{2}+w_{\eta }{\left\|\eta (u^{e})-Η\right\|}^{2} \end{array}$$

where the squared norms can be defined by the Euclidean norms as

(13a) $${\left\|e(u^{e})-Ε\right\|}^{2}\equiv \frac{1}{n}\iint_{A_{e}}\sum_{i=1}^{n}{\left[e{\left(u^{e}\right)}_{i}-{Ε}_{i}\right]}^{2}dx\;dy$$

(13b) $${\left\|\kappa (u^{e})-Κ\right\|}^{2}\equiv \frac{{\left(2h\right)}^{2}}{n}\iint_{A_{e}}\sum_{i=1}^{n}{\left[\kappa {\left(u^{e}\right)}_{i}-{Κ}_{i}\right]}^{2}dx\;dy$$

(13c) $${\left\|\mu^{\left(k\right)}(u^{e})-{Μ}^{j}\right\|}^{2}\equiv \frac{1}{n}\iint_{A_{e}}\sum_{i=1}^{n}{\left[\mu^{\left(k\right)}{\left(u^{e}\right)}_{i}-{Μ}_{i}^{j}\right]}^{2}dx\;dy$$

(13d) $${\left\|\gamma (u^{e})-\Gamma \right\|}^{2}\equiv \frac{1}{n}\iint_{A_{e}}\sum_{i=1}^{n}{\left[\gamma {\left(u^{e}\right)}_{i}-\Gamma_{i}\right]}^{2}dx\;dy$$

(13e) $${\left\|\eta (u^{e})-Η\right\|}^{2}\equiv \frac{1}{n}\iint_{A_{e}}\sum_{i=1}^{n}{\left[\eta {\left(u^{e}\right)}_{i}-{Η}_{i}\right]}^{2}dx\;dy$$

where the location of the embedded sensors (i.e., *j*-th interface, see Equation (13c)), can be any interface through the thickness coordinate of the laminate, such as j=k  or (k−1), where 1<k<N. In Equation (12), the symbol $w_{\alpha }\;(\alpha =e,\kappa ,\mu ,\gamma ,\eta )$ represents the vector of weighting constants corresponding to each individual section strain. Their usage is important for those problems involving few strain sensors. In the case of every analytic section, strain has a comparable experimental value; they can be set to unit vectors as:

(14) $$w_{\alpha }=\left[\begin{array}{ccc} 1 & 1 & 1 \end{array}\right]\;\;\;\;(\alpha =e,\kappa ,\mu ),\;\;w_{\beta }=\left[\begin{array}{cc} 1 & 1 \end{array}\right]\;\;\;\;(\beta =\gamma ,\eta )$$

On the other hand, if an experimental strain component is not available, the corresponding weighting constant can be set to a smaller number compared to unity, such as $\lambda =10^{-5}$, and the corresponding squared norm can be redefined by its reduced form as:

(15a) $${\left\|\chi (u^{e})\right\|}^{2}\equiv \iint_{A_{e}}{\left[\chi (u^{e})\right]}^{2}dx\;dy\;\;\;\;\;\;\;\;(\chi =e,\gamma ,\eta )$$

(15b) $${\left\|\kappa (u^{e})\right\|}^{2}\equiv {\left(2h\right)}^{2}\iint_{A_{e}}{\left[\kappa (u^{e})\right]}^{2}dx\;dy$$

(15c) $${\left\|\mu^{\left(k\right)}(u^{e})\right\|}^{2}\equiv \iint_{A_{e}}{\left[\mu^{\left(k\right)}(u^{e})\right]}^{2}dx\;dy$$

More information regarding the usage and importance of the weighting coefficients can be found in [43]. Note that all integrations in Equations (13) and (15) should be performed over the mid-plane area of an individual i3-RZT element, $A_{e}$. Finally, minimizing the $\Phi_{e}(u^{e})$ functional with respect to the $u^{e}$ nodal DOF reveals that

(16) $$\frac{\partial \Phi_{e}(u^{e})}{\partial u^{e}}=k^{e}u^{e}-\varepsilon^{e}=0\;\;\;\Rightarrow \;\;\;k^{e}u^{e}=\varepsilon^{e}$$

where the right-hand-side vector $\varepsilon^{e}$ is a function of the experimental strain values and can be defined as:

(17a) $$\begin{array}{l} \varepsilon^{e}=\frac{1}{n}\iint_{A_{e}}\sum_{i=1}^{n}(w_{e}{\left(B^{e}\right)}^{T}{Ε}_{i}+{\left(2h\right)}^{2}w_{\kappa }{\left(B^{\kappa }\right)}^{T}{Κ}_{i}+w_{\mu }{\left(B^{\mu }\right)}^{T}{\left(H_{\phi }^{\left(k\right)}\right)}^{T}{Μ}_{i}^{j}\\ \;\;\;\;\;\;\;\;\;\;\;\;\;\;\;\;\;+w_{\gamma }{\left(B^{\gamma }\right)}^{T}\Gamma_{i}+w_{\eta }{\left(B^{\eta }\right)}^{T}{Η}_{i})dx\;dy \end{array}$$

and where the element left-hand-side matrix $k^{e}$ is independent of the in situ strain data and can be defined as:

(17b) $$\begin{array}{l} k^{e}=\iint_{A_{e}}(w_{e}{\left(B^{e}\right)}^{T}B^{e}+{\left(2h\right)}^{2}w_{\kappa }{\left(B^{\kappa }\right)}^{T}B^{\kappa }+w_{\mu }{\left(B^{\mu }\right)}^{T}{\left(H_{\phi }^{\left(k\right)}\right)}^{T}H_{\phi }^{\left(k\right)}B^{\mu }\\ \;\;\;\;\;\;\;\;\;\;\;\;\;\;\;\;\;+w_{\gamma }{\left(B^{\gamma }\right)}^{T}B^{\gamma }+w_{\eta }{\left(B^{\eta }\right)}^{T}B^{\eta })dx\;dy \end{array}$$

An artificial contribution matrix, $k_{\psi }$, must be added to the $k^{e}$ matrix by providing the artificial stiffness terms associated with the drilling DOF of the zigzag amplitudes $\psi_{zi}\;(i=1,2,3)$. The $k_{\psi }$ matrix can be simply constructed as a diagonal form

(18a) $$k_{\psi }=\left[\begin{array}{ccc} k_{\psi z}^{1} & 0 & 0\\ 0 & k_{\psi z}^{2} & 0\\ 0 & 0 & k_{\psi z}^{3} \end{array}\right]$$

with

(18b) $$k_{\psi z}^{i}=10^{-5}\times \min(k_{\psi x}^{i},\;k_{\psi y}^{i})\;(i=1,2,3)$$

where the coefficients $k_{\psi x}^{i},\;k_{\psi y}^{i}\;(i=1,2,3)$ are diagonal terms of the $k^{e}$ matrix, corresponding to the zigzag-amplitude DOF, $\left(\psi_{xi},\;\psi_{yi})\;(i=1,2,3\right)$. Then, the element equations defined in Equation (16) can be rewritten in the following final form as:

(18c) $$\left[\begin{array}{cc} k_{v} & 0\\ 0 & k_{\psi } \end{array}\right]\left[\begin{array}{c} v\\ \psi_{z} \end{array}\right]=\left[\begin{array}{c} \varepsilon_{v}\\ 0 \end{array}\right]$$

where the $\psi_{z}$ vector denotes the drilling DOF of the zigzag amplitudes, the v vector represents all other DOFs of the i3-RZT element; the $k_{v}$ matrix and $\varepsilon_{v}$ vector are the sub-elements associated with the v vector.

Utilizing suitable transformation matrices, the element equations Equation (18c) can be transformed into global equations that can be subsequently assembled into the global system of matrix equations of the discretized structure. To disable rigid body modes of the discretized structure, the constraint boundary conditions need to be applied in accordance with standard FEM procedures. Finally, the solution of the global system of matrix equations will provide the total deformation (structural shape) of the structure at any real time.

## 3. Numerical Examples

**Figure 4:**
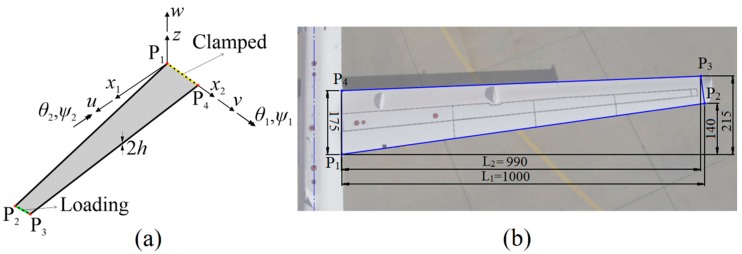
(**a**) Tapered wing-shaped sandwich plate; (**b**) dimensions [mm] of the plate, scaled in accordance with wing of NASA’s Ikhana vehicle [49].

In the remainder of this section, a tapered wing-shaped plate (sandwich laminate), depicted in Figure 4a, is considered to be analyzed based on the iFEM-RZT methodology. For the sake of consistency with an aircraft wing, the plate dimensions are scaled appropriately and fitted to the wing of NASA’s Ikhana aircraft, as shown in Figure 4b. The laminate has a uniform thickness of 2h=100  mm and span-to-thickness ratio of $L_{1}/2h=10$, thus representing a moderately thick plate. The laminate is seven-layer, quasi-isotropic sandwich laminate with carbon-epoxy (orthotropic material) face sheets and a thick closed cell polyvinyl chloride (PVC) core (isotropic material). As listed in Table 1 and Table 2, the mechanical properties of the carbon-epoxy and PVC are chosen as same as those given in [43] and the total stacking sequence is symmetric, with respect to the mid-plane of the laminate.

The yellow edge of the plate is fully clamped, as shown in Figure 4a, whereas the green edge is the loading edge, where surface tractions are applied in various directions to perform three main deformation states of the laminate, i.e., bending, torsion, and membrane deformations. First of all, a uniformly distributed force of 200 kN/m is applied in the negative z direction, to activate a bending state. Note that the bending load also creates some torsional deformations on the laminate, but the amount of torsional rotations is negligibly small, i.e., approximately one order of magnitude smaller than bending rotations. Therefore, the main deformation state of the laminate can be referred to as the bending state. Secondly, the torsion scenario is performed by applying a uniformly distributed moment (torque) of 40 kNm/m, in the positive $x_{1}$ axis. Finally, the loading edge is subjected to a uniformly distributed force of 1000 kN/m, in the positive $x_{2}$ direction, for triggering the membrane behavior of the plate. 

**Figure 5:**
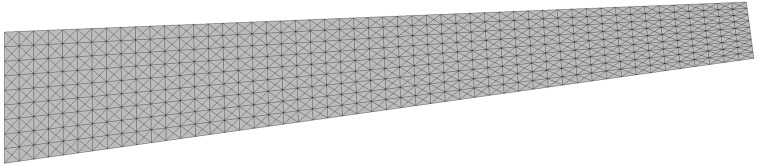
High-fidelity discretization, consisting of 1836 uniformly distributed triangular elements and possessing 8811 degrees of freedom (DOF).

Prior to the shape sensing analysis of the tapered plate, we performed a series of RZT-based finite element analyses (convergence study), utilizing a three-node triangular plate element, known as an RZT *constrained anisoparametric* plate element [50], having seven DOF per node. The high-fidelity discretization, shown in Figure 5, provides sufficiently accurate (convergent) displacements and rotations that can be used as reference solutions for the loading cases mentioned above. These FEM analyses are also utilized to generate the discrete strain-sensor data that simulates experimental strain readings obtained from surface patched strain gauges and embedded FBG sensors. 

**Figure 6:**
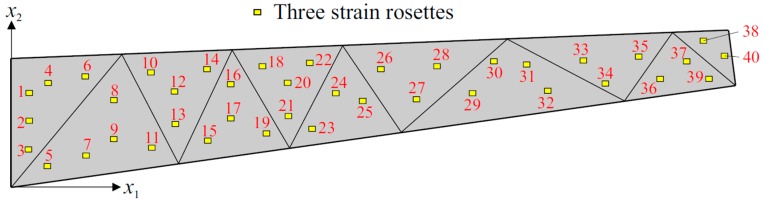
Dense (D) sensor placement configuration with 40 × 3 strain rosettes.

**Figure 7:**
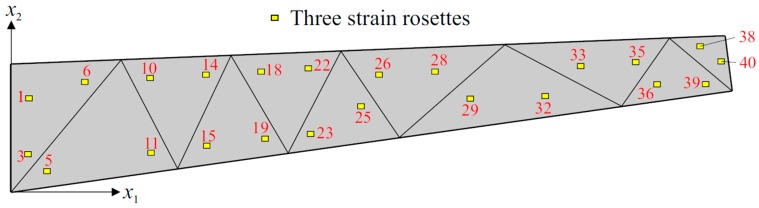
Sparse (S) sensor placement configuration with 23 × 3 strain rosettes.

**Figure 8:**
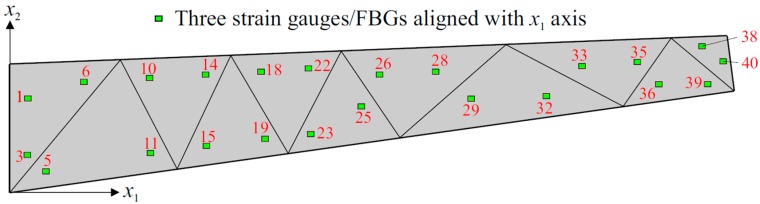
Very Sparse (VS) sensor placement configuration with 23 × 3 fiber Bragg grating (FBG) sensors.

**Figure 9:**
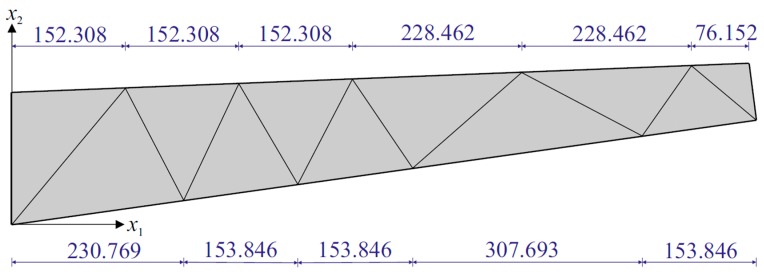
Dimensions [mm] of smoothing element analysis (SEA) domain consisted of 11 triangular elements.

Three different sensor placement configurations, depicted in Figure 6, Figure 7 and Figure 8, are considered in the following shape sensing analysis. The exact in-plane coordinates of each strain sensor location are listed in Table 3. Regarding the through-the-thickness coordinates of these sensors, they are located at three different interfaces of the laminate: the top surface (z=+h), the bottom surface (z=−h), and the first interface among those of the bottom face sheets (z=−0.8  h). The density (total number) of strain data sampling is different for each sensor network; thus, we distinguish these configurations from each other by referring to them as “Dense (D)”, “Sparse (S)” and “Very Sparse (VS)”, respectively (Figure 6, Figure 7 and Figure 8). Triaxial strains are measured by 40  ×  3 and 23  ×  3 strain rosettes for the D and S networks, respectively, whereas uniaxial strain measurements are made by 23  ×  3 FBG sensors for the VS network. Therefore, the total number of strain data sampling for the D, S and VS sensor placement configurations count as 120 × 3 = 360, 69 × 3 = 207, and 69 × 1 = 69, respectively. Thanks to triaxial strain measurements, experimental section strains at each specific in situ location can be computed independently from the direction (orientation) of the sensors for both D and S configurations. However, the alignment of sensors plays an important role for the VS configuration, since the uniaxial strain measurements allow us to compute only one experimental section strain, which is along the FBG sensor direction. For each sensor placement configuration, the smoothing element analysis is employed, utilizing a smoothing domain, composed of eleven triangular elements whose dimensions are clearly shown in Figure 9. In this regard, discretely measured surface strains are computed as C^1^-continuous polynomial functions of in-plane axes, $x_{\alpha }\;(\alpha =1,2)$. Therefore, the iFEM analysis, corresponding to each sensor placement model, can be performed using any fine i3-RZT mesh. Although it is not a limitation, for simplicity, we chose to use the high-fidelity discretization, depicted in Figure 5 as the fine i3-RZT mesh, in the following iFEM-RZT analysis.

During the iFEM-RZT analysis, the weighting constants associated with the membrane, bending, and zigzag strain measures are adjusted to unity for configurations D and S as:

(19a) $$w_{\alpha }=\left[\begin{array}{ccc} 1 & 1 & 1 \end{array}\right]\;\;\;\;(\alpha =e,\kappa ,\mu )$$

On the other hand, each strain gauge/FBG sensor depicted in Figure 8 is aligned with the $x_{1}$ axis for the VS configuration. Therefore, while performing the shape sensing analyses with this model, the weighting coefficients can be adjusted as:

(19b) $$w_{\alpha }=\left[\begin{array}{ccc} 1 & \lambda & \lambda \end{array}\right]\;\;\;\;(\alpha =e,\kappa ,\mu )$$

where weighting constants corresponding to the strain measures (membrane, bending and zigzag) along the $x_{2}$ axes and in the $x_{1}x_{2}$ plane are set to a small value, $\lambda =10^{-3}$, since the FBG/strain gauges sensors can provide strain measurements along the $x_{1}$ direction only. To maintain the accuracy of rotation predictions, while at the same time increasing the quality of deflection predictions, the weighting constants associated with the transverse-shear strains are also set to small values for all sensor placement configurations. It is important to note that the deformation of quasi-isotropic laminate produces much greater values of first transverse-shear strains ($\gamma (u^{e})$) than those of second transverse-shear strains ($\eta (u^{e})$). Therefore, the following adjustment of weighting constants corresponding to transverse-shear strain measures are made for all three models:

(20) $$w_{\gamma }=\left[\begin{array}{cc} \lambda_{1} & \lambda_{1} \end{array}\right]\;,\;\;w_{\eta }=\left[\begin{array}{cc} \lambda_{2} & \lambda_{2} \end{array}\right]$$

where $\lambda_{1}=10^{-8}$ takes on a smaller value than $\lambda_{2}=10^{-4}$, thus ensuring the necessary strain compatibility between the first and second transverse-shear measures of the sandwich laminate. 

**Figure 10:**
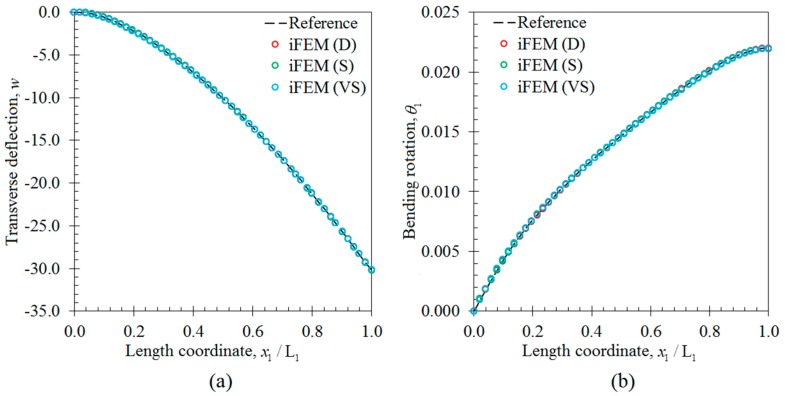
Bending variations along the $x_{1}$ direction on the edge between points P_1_ and P_2_: (**a**) transverse deflection w [mm]; (**b**) the bending rotation $\theta_{1}$ [rad].

**Figure 11:**
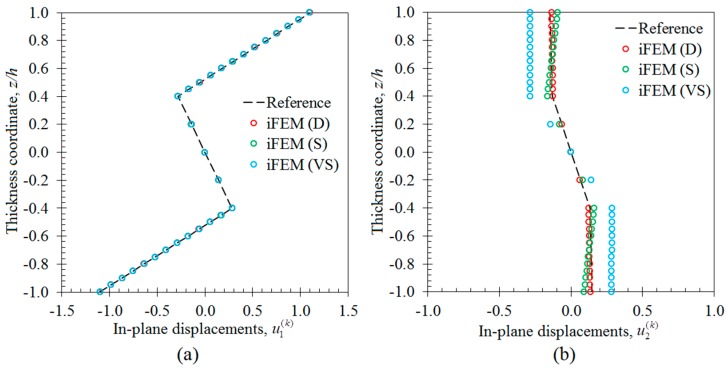
Interfacial displacement variations through the thickness of laminate at point P_2_ for bending scenario: (**a**) $u_{1}^{\left(k\right)}(1000,140,z)$ [mm]; (**b**) $u_{2}^{\left(k\right)}(1000,140,z)$ [mm].

**Figure 12:**
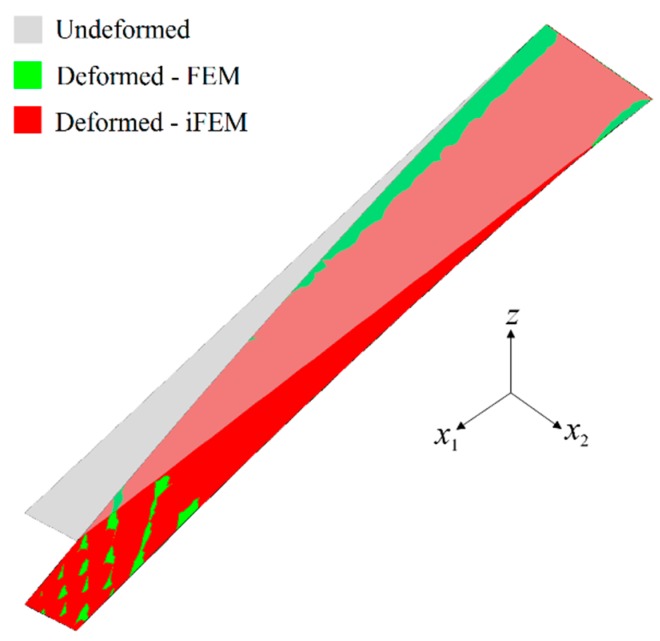
Three-dimensional shape sensing for bending scenario: comparison between the high-fidelity FEM analysis and iFEM analysis of VS configuration.

In the first case study, the iFEM analysis was performed by using the discrete strain data obtained from the bending scenario. The transverse deflection w and bending rotation $\theta_{1}$ variations obtained from the iFEM analysis were compared with those of reference high-fidelity FEM analysis, as depicted in Figure 10, respectively. These results demonstrate that all three sensor placement configurations enable the prediction of accurate bending responses of the sandwich plate. In addition, the through-the-thickness variation of the interfacial displacements, $u_{1}^{\left(k\right)}$ and $u_{2}^{\left(k\right)}$, at point P_2_, are plotted in Figure 11. These variations show that all three models produce a zigzag interfacial displacement of $u_{1}^{\left(k\right)}$ that is indistinguishable from their reference values. On the other hand, although the i3-RZT model, corresponding to the VS configuration, predicts erroneous $u_{2}^{\left(k\right)}$ displacements, they will not affect the computation of the three-dimensional deformed shape, as their maximum values are one order of magnitude lower than those of $u_{1}^{\left(k\right)}$ displacements. As depicted in Figure 12, the deformed shape of the sandwich laminate, predicted using the i3-RZT model of VS configuration, has a perfect match with the one produced by the FEM analysis. Remarkably, iFEM-RZT methodology can estimate a highly accurate deformed shape for the bending scenario, even if the model containing the sparsest strain measurements is used in the analysis. Hence, only a very sparse longitudinal strain data is sufficient for predicting accurate full-field bending deformations, including a true zigzag representation of interfacial displacements.

**Figure 13:**
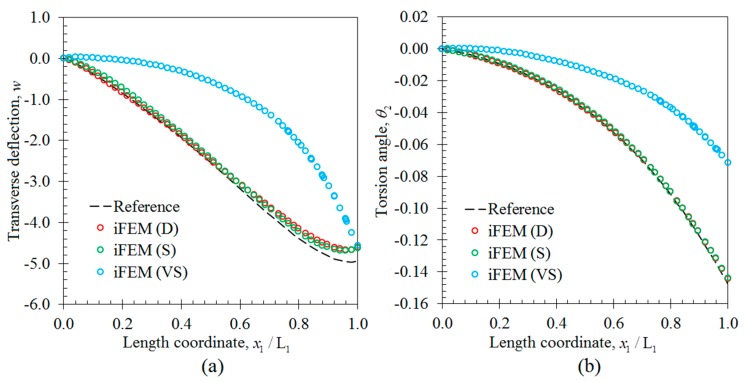
Torsion variations along the $x_{1}$ direction on the edge between points P_1_ and P_2_: (**a**) the transverse deflection w [mm]; (**b**) the torsion angle $\theta_{2}$ [rad].

**Figure 14:**
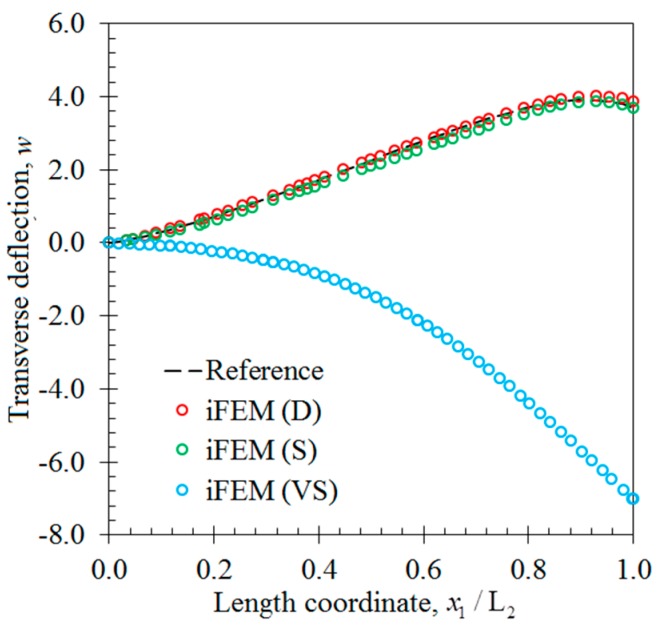
The transverse deflection, w [mm], variation along $x_{1}$ direction on the edge between points P_3_ and P_4_: torsion scenario of the laminate.

**Figure 15:**
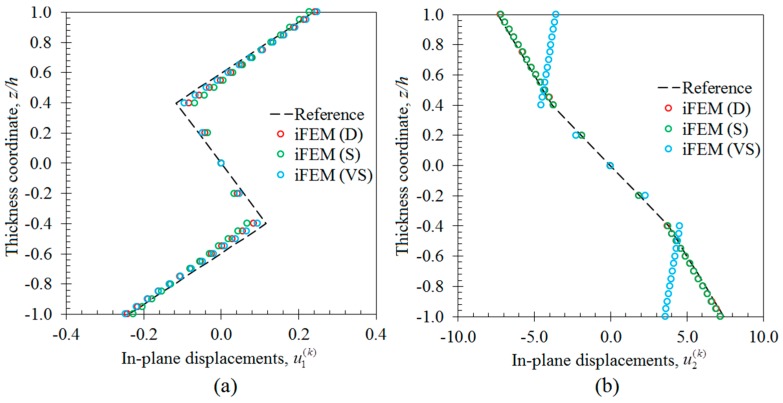
Interfacial displacement variations through the thickness of laminate at point P_2_ for the torsion scenarios: (**a**) $u_{1}^{\left(k\right)}(1000,140,z)$ [mm]; (**b**) $u_{2}^{\left(k\right)}(1000,140,z)$ [mm].

**Figure 16:**
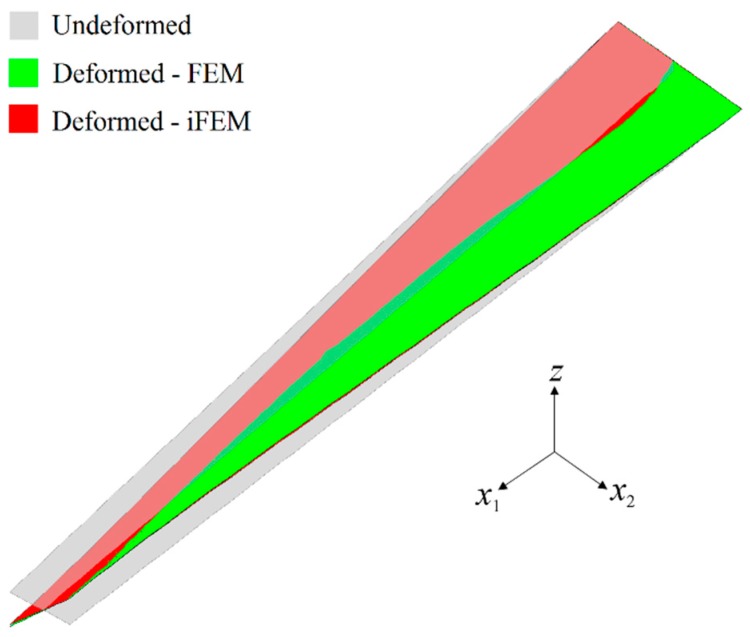
Three-dimensional shape sensing for torsion scenario: comparison between the high-fidelity FEM analysis and iFEM analysis of the S configuration.

In the second case study, the iFEM analysis was carried out using i3-RZT models, which contained the discrete strain measurements obtained from the torsion scenario. The variations of transverse deflection (w) and the torsion angle ($\theta_{2}$) on the edge (P_1_–P_2_) are plotted in Figure 13. These results clearly illustrate that both i3-RZT models corresponding to D and S sensor placement configurations can attain accurate deflection results that underestimate their maximum reference values by less than 5%. Also, they can predict $\theta_{2}$ torsion rotations almost as same as those of FEM analysis. However, the VS configuration that contains only uniaxial strain measurements could not produce precise enough results for both w and $\theta_{2}$ torsion variables. In fact, due to the lack of experimental shear strain measurements, this model aims to predict a bending response rather than a torsion response. This fact is clearly depicted in Figure 14 by plotting the variations of transverse deflection, w, on the edge, P_3_–P_4_. This deflection should be positive for the torsion scenario, although the i3-RZT model corresponding to VS configuration predicts negative (erroneous) deflections. Nevertheless, both D and S configurations produced highly accurate and positive deflections, that correlated very well with the reference solution of torsion scenario, as depicted in Figure 14. In addition, the in-plane displacements, $u_{1}^{\left(k\right)}$ and $u_{2}^{\left(k\right)}$, produced by both D and S models, exhibited a highly zigzagged distribution through the thickness coordinate, which are well in agreement with their reference solutions, as presented in Figure 15. In contrast, the VS model produced an inaccurate evaluation of the $u_{2}^{\left(k\right)}$ displacements, compared to the reference solution in Figure 15b, although it predicts a sufficiently accurate $u_{1}^{\left(k\right)}$ displacement in Figure 15a. Therefore, only a dense or sparse distribution of sensors that measures triaxial strains at each particular in situ location, can perform a true reconstruction of the three-dimensional deformed shapes. For example, the deformed shape, obtained using the S sensor placement configuration, is virtually indistinguishable from those of the reference deformed shape in Figure 16. Hence, it is confirmed that a sparse deployment of strain rosettes is sufficient to use in the iFEM-RZT analysis for performing a highly accurate shape sensing of torsional displacements. 

**Figure 17:**
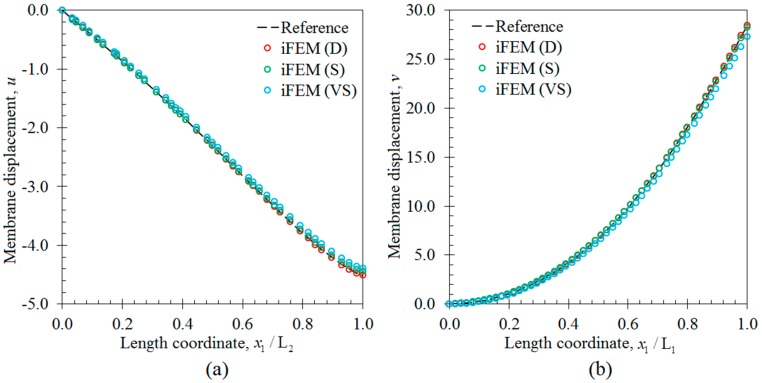
Membrane deformation variations along the $x_{1}$ direction: (**a**) the membrane displacement u [mm] on the edge between points P_3_ and P_4_; (**b**) the membrane displacement v [mm] on the edge between points P_1_ and P_2_.

**Figure 18:**
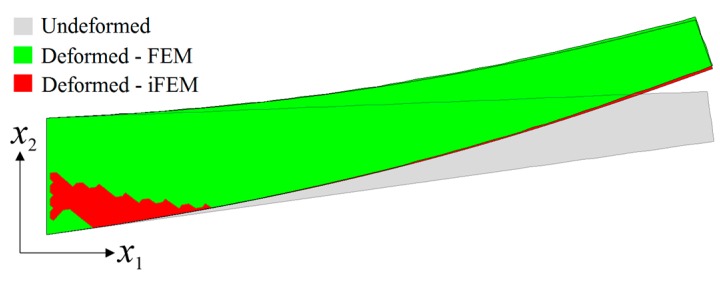
Three-dimensional shape sensing for the membrane deformation scenario: comparison between high-fidelity FEM analysis and iFEM analysis of VS configuration.

In the third case study, all three sensor placement configurations were used to perform the shape sensing of the membrane deformation scenario of the laminate. In contrast to bending and torsion scenarios, the internal sensors do not necessarily need to be embedded inside the laminate, since the top and bottom surface strain measurements can be directly used to account for membrane displacements, based on iFEM methodology. Therefore, for the following analysis, the embedded sensors were removed and the total number of strain data sampling was reduced to 240, 138 and 46 for the D, S and VS sensor placement configurations, respectively. The i3-RZT models of each sensor network can estimate precise variations of u and v membrane displacements, that agree very well with the reference solutions in Figure 17. Therefore, a sparse distribution of FBG sensors can perform very accurate shape sensing of a membrane scenario, which is as accurate as those predicted by a dense distribution of strain rosettes. The deformed shape produced by VS configuration is almost as same as from those with the reference shape in Figure 18, demonstrating the superior practical capability of iFEM framework for the membrane scenario. Overall, the potential and versatile applicability of iFEM-RZT methodology is demonstrated for three-dimensional shape sensing of future aerospace structures which may be subjected to various loads, including bending, torsion, and membrane conditions.

## 4. Conclusions 

Shape sensing of a tapered wing-shaped thick sandwich plate is performed for membrane, bending, and torsion loading conditions. Three different sensor placement configurations were analyzed, based on an enhanced iFEM-RZT formulation, in order to investigate the effect of varying strain data density and sensor alignment on the shape-sensing accuracy corresponding to each loading condition. The iFEM-RZT formulation uses only the discretely measured strains and minimizes a weighted-least-squares functional, that involves the complete set of strain measures consistent with a state-of-the-art RZT plate theory. Thus, it presents the following advantages: (1) the requirement of only few strain sensors; (2) suitability to develop C°-continuous inverse-shell elements (e.g., i3-RZT); and (3) accurate modelling of interfacial displacement variations through thickness. The iFEM analysis of the wing-shaped plate demonstrated that the i3-RZT model of a sparse sensor placement configuration can predict bending, torsion, and membrane deformed shapes, with an accuracy level that is very similar to those of predicted by a dense distribution of sensors. Therefore, the computational efficiency, high accuracy, and robustness of the i3-RZT element is valid and the element formulation is suitable for shape sensing of the membrane-bending coupled structural responses, including the torsion phenomenon. 

In particular, it was observed that a sparse distribution of FBG sensors aligned with only the longitudinal direction is capable of predicting accurate full-field bending and membrane deformations. However, the same sparse model with uniaxial strains was not able to reconstruct true torsional displacements, since the experimental shear strain measurements have a significant contribution to torsion deformations. Thus, usage of strain rosettes (triaxial strains) is necessary and it was demonstrated that only a sparse deployment of rosettes is sufficient to capture highly accurate three-dimensional torsional shapes. Note that the shape sensing of membrane deformations does not require the embedding of sensors inside the composite/sandwich structure, while it is necessary for the shape sensing of bending and torsion deformations, especially for modeling precise zigzag distribution of interfacial displacements. Finally, it can be concluded that the present iFEM-RZT analysis of the sandwich plate contributes to a potential sensor placement model for shape sensing and real-time structural health monitoring of future aerospace vehicles.

## Figures and Tables

**Table 1:** Mechanical properties of orthotropic and isotropic materials.

Lamina Material		Young’s Modulus [GPa]	Poisson’s Ratio	Shear Modulus [GPa]
C	Carbon-epoxy unidirectional composite	$\begin{array}{l} E_{1}^{\left(k\right)}=157.9\\ E_{2}^{\left(k\right)}=9.584\\ E_{3}^{\left(k\right)}=9.584 \end{array}$	$\begin{array}{l} \upsilon_{12}^{\left(k\right)}=0.32\\ \upsilon_{13}^{\left(k\right)}=0.32\\ \upsilon_{23}^{\left(k\right)}=0.49 \end{array}$	$\begin{array}{l} G_{12}^{\left(k\right)}=5.930\\ G_{13}^{\left(k\right)}=5.930\\ G_{23}^{\left(k\right)}=3.227 \end{array}$
P	PVC core	$E^{\left(k\right)}=0.104$	$\upsilon^{\left(k\right)}=0.3$	$G^{\left(k\right)}=0.04$

**Table 2:** Laminate stacking sequence in the positive z direction.

Laminate	Normalized Lamina Thickness, $h^{\left(k\right)}/h$	Lamina Materials	Lamina Orientation [°]
Quasi-isotropic sandwich	(0.1/0.1/0.1/0.4/0.1/0.1/0.1)	(C/C/C/P/C/C/C)	(60/0/−60/0/−60/0/60)

**Table 3:** In-plane coordinates of sensor locations.

Sensors	*x*_1_ [mm]	*x*_2_ [mm]	Sensors	*x*_1_ [mm]	*x*_2_ [mm]
1	22.71	127.94	21	381.15	96.59
2	22.76	89.56	22	411.57	169.35
3	22.81	51.18	23	414.34	80.35
4	48.63	142.31	24	445.47	127.79
5	48.96	28.91	25	485.03	117.29
6	100.46	151.57	26	509.37	161.16
7	101.14	41.66	27	556.86	120.15
8	139.66	118.03	28	587.23	166.48
9	140.13	64.38	29	635.01	128.37
10	191.20	156.76	30	665.10	171.80
11	192.49	52.95	31	711.05	167.24
12	224.03	130.38	32	739.77	131.86
13	224.70	85.21	33	788.97	173.13
14	268.98	161.20	34	817.98	140.66
15	270.79	62.62	35	866.89	179.03
16	301.84	141.63	36	896.20	149.46
17	302.85	93.43	37	933.22	171.91
18	346.76	165.65	38	956.02	200.46
19	349.09	72.29	39	962.45	148.07
20	379.88	142.17	40	984.88	179.43

## Appendix A

The shape functions, $N_{i}$, $M_{i}$ and $L_{i}$, that defined both membrane and bending capability of i3-RZT element as given in Equations (1) and (2), can be respectively defined as:

(A1) $$N_{i}=\frac{b_{i}x_{1}+a_{i}x_{2}+c_{i}}{2A_{e}}$$

(A2) $$M_{i}=\frac{N_{i}}{2}(a_{k}N_{j}-a_{j}N_{k})$$

(A3) $$L_{i}=\frac{N_{i}}{2}(b_{k}N_{j}-b_{j}N_{k})$$

with

(A4) $$a_{i}=x_{1}^{k}-x_{1}^{j}$$

(A5) $$b_{i}=x_{2}^{j}-x_{2}^{k}$$

(A6) $$c_{i}=x_{1}^{j}x_{2}^{k}-x_{1}^{k}x_{2}^{j}$$

where the symbol, $x_{\alpha }^{i}\;\;(\alpha =1,2)$, denotes the local coordinates of element nodes and the subscripts/superscripts vary as j=2,3,1 and k=3,1,2 for node numbers i=1,2,3.

Moreover, the matrix of shape function derivatives, $B^{\alpha }\;\;(\alpha =e,\kappa ,\mu ,\gamma ,\eta )$, given in Equation (8a–e) can be expressed as:

(A7) $$B^{\alpha }=\left[\begin{array}{ccc} B_{1}^{\alpha } & B_{2}^{\alpha } & B_{3}^{\alpha } \end{array}\right]\;(\alpha =e,\kappa ,\mu ,\gamma ,\eta )$$

with

(A8) $$B_{i}^{e}=\left[\begin{array}{ccccccccc} N_{i,1} & 0 & 0 & 0 & 0 & L_{i,1} & 0 & 0 & 0\\ 0 & N_{i,2} & 0 & 0 & 0 & M_{i,2} & 0 & 0 & 0\\ N_{i,2} & N_{i,1} & 0 & 0 & 0 & M_{i,1}+L_{i,2} & 0 & 0 & 0 \end{array}\right]$$

(A9) $$B_{i}^{\kappa }=\left[\begin{array}{ccccccccc} 0 & 0 & 0 & 0 & N_{i,1} & 0 & 0 & 0 & 0\\ 0 & 0 & 0 & -N_{i,2} & 0 & 0 & 0 & 0 & 0\\ 0 & 0 & 0 & -N_{i,1} & N_{i,2} & 0 & 0 & 0 & 0 \end{array}\right]$$

(A10) $$B_{i}^{\mu }=\left[\begin{array}{ccccccccc} 0 & 0 & 0 & 0 & 0 & 0 & 0 & N_{i,1} & 0\\ 0 & 0 & 0 & 0 & 0 & 0 & -N_{i,2} & 0 & 0\\ 0 & 0 & 0 & 0 & 0 & 0 & 0 & N_{i,2} & 0\\ 0 & 0 & 0 & 0 & 0 & 0 & -N_{i,1} & 0 & 0 \end{array}\right]$$

(A11) $$B_{i}^{\gamma }=\left[\begin{array}{ccccccccc} 0 & 0 & N_{i,1} & -L_{i,1} & N_{i}-M_{i,1} & 0 & L_{i,1} & M_{i,1} & 0\\ 0 & 0 & N_{i,2} & -L_{i,2}-N_{i} & -M_{i,2} & 0 & L_{i,2} & M_{i,2} & 0 \end{array}\right]$$

(A12) $$B_{i}^{\eta }=\left[\begin{array}{ccccccccc} 0 & 0 & N_{i,1} & -L_{i,1} & N_{i}-M_{i,1} & 0 & L_{i,1} & M_{i,1}-N_{i} & 0\\ 0 & 0 & N_{i,2} & -L_{i,2}-N_{i} & -M_{i,2} & 0 & L_{i,2}+N_{i} & M_{i,2} & 0 \end{array}\right]$$

where the subscripts vary as i=1,2,3.
